# An evaluation of the metabolic syndrome in a large multi-ethnic study: the Family Blood Pressure Program

**DOI:** 10.1186/1743-7075-2-17

**Published:** 2005-08-02

**Authors:** Aldi T Kraja, DC Rao, Alan B Weder, Thomas H Mosley, Stephen T Turner, Chao Agnes Hsiung, Thomas Quertermous, Richard Cooper, J David Curb, Michael A Province

**Affiliations:** 1 Division of Biostatistics, Washington University School of Medicine, St. Louis, MO, USA; 2 University of Michigan Hospitals, Ann Arbor, MI, USA; 3 Division of Geriatrics, Department of Medicine, University of Mississippi Medical Center, Jackson MS, USA; 4 Mayo Clinic, College of Medicine, Rochester, MN, USA; 5 National Health Research Institutes, Division of Biostatistics, Taipei, Taiwan; 6 Stanford University School of Medicine, Stanford, CA, USA; 7 Loyola University Medical Center, Maywood, IL, USA; 8 Pacific Health Research Institute, Honolulu, HI, USA

## Abstract

**Background:**

The Family Blood Pressure Program is an ongoing, NHLBI-sponsored, multi-center program to study the genetic determinants of high blood pressure. The goal of this particular study was to study patterns of metabolic syndrome (MetS) in four ethnic groups: African Americans, Caucasians, Hispanics, and Asians.

**Methods:**

A major part of participants in three networks GENOA, HyperGEN and SAPPHIRe were recruited mainly through hypertensive probands. MetS was defined as a categorical trait following the National Cholesterol Education Program definition (c-MetS). MetS was also characterized quantitatively through multivariate factor analyses (FA) of 10 risk variables (q-MetS). Logistic regression and frequency tables were used for studying associations among traits.

**Results:**

Using the NCEP definition, the Hispanic sample, which by design was enriched for type 2 diabetes (T2D), had a very high prevalence of MetS (73%). In contrast, its prevalence in Chinese was the lowest (17%). In African Americans and Hispanics, c-MetS was more prevalent in women than in men. Association of c-MetS with type 2 diabetes (T2D) was prominent in the Hispanics and African Americans, less pronounced in the Whites and Japanese, (although still significant), and weakest in the Chinese sample.

Using FA without rotation, we found that the main factor loaded obesity (OBS) and blood pressure (BP) in African Americans; OBS and insulin (INS) in Hispanics, in Japanese, and in Whites; and OBS alone in Chinese. In Hispanics, Whites, and Japanese, BP loaded as a separate factor. Lipids in combination with INS also loaded in a separate factor. Using FA with Varimax rotation, 4 independent factors were identified: "Obesity-INS," "Blood pressure," "Lipids-INS," and "Central obesity." They explained about 60% of the variance present in the original risk variables.

**Conclusion:**

MetS ethnic differences were identified. Ascertaining for hypertension or T2D increased the MetS prevalence in networks compared with the one in the US general population. Obesity was the most prominent risk factor contributing to both c-MetS and q-MetS. INS contributed in two important factors (obesity and lipids). The information imbedded into c-MetS trait /q-MetS factors scores can contribute in future research of the MetS, especially its utilization in the genetic analysis.

## Background

Metabolic syndrome (MetS) is defined as a clustering of cardiovascular and type 2 diabetes risk factors including obesity, insulin resistance, dyslipidemia, and hypertension (HT). The genetic control of MetS is expected to be complex since it represents a syndrome of multifaceted abnormalities. Categorical and clinically applicable criteria were developed by the National Cholesterol Education Program (NCEP), which defined MetS as the presence in an individual of at least 3 out of 5 risk factors (increased waist circumference (WAIST), increased level of triglycerides (TG), low levels of high density lipoprotein cholesterol (HDL), HT, and fasting glucose (GLUC) ≥ 110 mg/dl) [1].

We designated this dichotomous definition of MetS (presence or absence) as c-MetS (see also Material and Methods). Quantitative factor analytic treatment of MetS was designated as q-MetS (see Material and Methods). Several earlier studies have employed multivariate techniques such as factor analysis (FA) to investigate MetS. This method transforms a set of MetS risk variables to a smaller set of latent factors. Most studies have reported 2 to 4 underlying factors, depending on the number of risk factors included, whether or not the Varimax rotation was used, and statistical decisions made [2-7].

This study is an investigation of MetS in the Family Blood Pressure Program (FBPP) [8]. The FBPP is comprised of 4 different networks: GenNet, GENOA, HyperGEN, and SAPPHIRe. These networks were established to study the genetic determinants of high blood pressure.

The goal of this particular study was to evaluate MetS in the rich FBPP database using both the c-MetS and q-MetS definitions. Common features and differences among the major ethnic groups were explored. Finally, the relationships of c-MetS with T2D and vascular heterogeneous atherosclerotic (VHA) events were also investigated.

## Materials and methods

### Participants

The **FBPP **pooled database (version 3) of 13,592 participants from 4 different networks represents one of the largest compilations of ethnically diverse data. **GenNet **had only partial data for defining c-MetS and therefore, data from GenNet were excluded from analysis. **GENOA **(Genetic Epidemiology Network of Atherosclerosis) includes 3 field centers: the Jackson, MS center recruited African Americans; the one in Starr County, TX recruited Hispanics; the one in Rochester, MN, recruited Whites. **HyperGEN **(Hypertension Genetic Epidemiology Network) included field centers in Birmingham AL, which recruited African Americans; the rest of centers in Forsyth County, NC, in Framingham, MA, in Minneapolis, MN, and in Salt Lake City, UT recruited Whites. **SAPPHIRe **(Stanford Asian Pacific Program in Hypertension and Insulin Resistance), with 3 major field centers, recruited Asian Pacific populations of Chinese origin residing in Taiwan and of Japanese origin residing in Hawaii, and California. GENOA recruited African American, Hispanic, and White sibships with at least 2 hypertensive sibs (with HT onset before the age of 60). The Hispanic sibships were recruited with at least 2 sibs who were each diagnosed as type 2 diabetic (T2D). HyperGEN recruited African American and White hypertensive sibships with 2 or more hypertensive sibs, at least 1 of them having severe HT. In addition, HyperGEN recruited random samples of African American and White participants, and parents. SAPPHIRe recruited sib-pairs concordant and/or discordant for hypertension. For all participants the diet was uncontrolled and reflective of the "free-living" dietary habits of these populations.

We analyzed data from the FBPP where participants with data for defining c-MetS included: 1857 African Americans, 1799 Hispanics, and 1578 Whites in GENOA; 2010 African Americans, and 1888 Whites in HyperGEN; 1630 Chinese, and 581 Japanese participants in SAPPHIRe). In all networks, subjects with unknown ethnicity were excluded. As a result, information on a total of 11,343 participants was considered in the c-MetS study (Tables 1, 2, 3). In contrast, the sample sizes for q-MetS were considerably smaller because there were only 7,562 participants with no missing values for any of the 10 risk factors (see Material and Methods for risk factors analyzed and Table 5 for the exact sample sizes).

**Table 1:** Variables Analyzed in the FA (GENOA)

	**African Americans (N = 1312)**		**Hispanics (1160)**		**Whites (1073)**	
**Variables**	**Mean**	**St. Dev.**	**Mean**	**St. Dev.**	**Mean**	**St. Dev.**
**AGE**	58	10	55	12	55	11
**BMI**	31	7	31	6	30	6
**WAIST**	103	17	107	14	99	16
**WHR**	0.91	0.08	0.97	0.08	0.91	0.09
**INS**	11	10	14	14	9	7
**GLUC**	108	42	142	64	98	26
**LDL**	121	40	116	35	122	34
**HDL**	57	18	47	13	54	16
**TG**	126	43	162	47	156	47
**SBP**	130	23	129	22	133	19
**DBP**	71	11	70	10	76	10

**Table 2:** Variables Analyzed in the FA (HyperGEN)

	**African Americans (1731)**		**Whites (1255)**	
**Variables**	**Mean**	**St. Dev.**	**Mean**	**St. Dev.**
**AGE**	48	13	56	13
**BMI**	32	7	30	6
**WAIST**	102	17	103	15
**WHR**	0.90	0.08	0.94	0.08
**INS**	11	9	8	6
**GLUC**	109	45	103	30
**LDL**	120	37	118	32
**HDL**	54	15	49	15
**TG**	104	56	152	75
**SBP**	130	22	123	20
**DBP**	75	12	69	11

**Table 3:** Variables Analyzed in the FA (SAPPHIRe)

	**Chinese (747)**		**Japanese (284)**	
**Variables**	**Mean**	**St. Dev.**	**Mean**	**St. Dev.**
**AGE**	50	9	55	9
**BMI**	25	3	27	4
**WAIST**	84	11	90	12
**WHR**	0.87	0.08	0.91	0.09
**INS**	8	5	8	5
**GLUC**	92	18	101	21
**LDL**	124	37	121	34
**HDL**	43	12	48	14
**TG**	124	69	166	78
**SBP**	132	25	136	21
**DBP**	78	14	79	11

**Table 5:** Correlation Matrices Among Risk Factors Prepared for MetS by Network

**GENOA**	**N = 1160**	**Hispanics**									
**N = 1312**		**BMI**	**WAIST**	**WHR**	**INS**	**GLUC**	**LDL**	**HDL**	**TG**	**SBP**	**DBP**
**A.**	**BMI**	-	0.84‡	0.33‡	0.40‡	-0.15‡	-0.03	-0.17‡	0.12‡	0.16‡	0.03
**A**	**WAIST**	0.87‡	-	0.63‡	0.37‡	-0.19‡	-0.03	-0.18‡	0.12‡	0.19‡	0.06*
**m**	**WHR**	0.38‡	0.68‡	-	0.24‡	-0.21‡	0.03	-0.16‡	0.13‡	0.18‡	0.10‡
**e**	**INS**	0.45‡	0.45‡	0.31‡	-	-0.13‡	-0.08†	-0.18‡	0.19‡	0.10	0.03
**r**	**GLUC ††**	-0.28‡	-0.30‡	-0.26‡	-0.38‡	-	-0.03	0.15‡	-0.18‡	-0.17‡	-0.10‡
**I**	**LDL**	0.03	0.04	0.05	0.05	-0.03	-	0.01	0.19‡	0.04	0.07*
**c**	**HDL**	-0.18‡	-0.21‡	-0.20‡	-0.33‡	0.18‡	-0.12	-	-0.30‡	0.01	0.05
**a**	**TG**	0.13‡	0.19‡	0.21‡	0.30‡	-0.20‡	0.20‡	-0.34‡	-	0.14‡	0.09†
**n**	**SBP**	0.13‡	0.14‡	0.14‡	0.08†	-0.12‡	0.04	0.00	0.06*	-	0.72‡
**s**	**DBP**	-0.02	0.00	0.06*	-0.01	-0.02	0.06*	0.03	0.04	0.75‡	-

**GENOA**											

**N = 1073**		**BMI**	**WAIST**	**WHR**	**INS**	**GLUC**	**LDL**	**HDL**	**TG**	**SBP**	**DBP**

	**BMI**	-									
	**WAIST**	0.90‡	-								
	**WHR**	0.51‡	0.74‡	-							
	**INS**	0.57‡	0.57‡	0.42‡	-						
**W**	**GLUC**	-0.32‡	-0.31‡	-0.24‡	-0.41‡	-					
**h**	**LDL**	0.00	0.02	0.01	0.00	-0.02	-				
**i**	**HDL**	-0.28‡	-0.30‡	-0.27‡	-0.37‡	0.18‡	-0.12	-			
**t**	**TG**	0.28‡	0.29‡	0.21‡	0.31‡	-0.17‡	0.16‡	-0.37‡	-		
**e**	**SBP**	0.24‡	0.22‡	0.14‡	0.16‡	-0.15‡	0.00	-0.04	0.12‡	-	
**s**	**DBP**	0.07*	0.09†	0.09†	0.03	-0.02	0.05	0.02	0.12‡	0.68‡	-

**HyperGEN**	**N = 1255**	**Whites**									
**N = 1731**		**BMI**	**WAIST**	**WHR**	**INS**	**GLUC**	**LDL**	**HDL**	**TG**	**SBP**	**DBP**

**A.**	**BMI**	-	0.89‡	0.45‡	0.51‡	-0.31‡	0.04	-0.19‡	0.22‡	0.17‡	0.01
**A**	**WAIST**	0.90‡	-	0.68‡	0.50‡	-0.32‡	0.06*	-0.19‡	0.24‡	0.13‡	0.00
**m**	**WHR**	0.45‡	0.70‡	-	0.35‡	-0.24‡	0.06*	-0.21‡	0.25‡	0.12‡	0.07*
**e**	**INS**	0.49‡	0.51‡	0.43‡	-	-0.30‡	-0.03	-0.35‡	0.35‡	0.17‡	0.07*
**r**	**GLUC**	-0.27‡	-0.30‡	-0.29‡	-0.40‡	-	-0.05	0.19‡	-0.21‡	-0.13‡	-0.02
**I**	**LDL**	0.12‡	0.11‡	0.09‡	0.09‡	-0.08	-	-0.01	0.08†	0.05	0.06*
**c**	**HDL**	-0.21‡	-0.24‡	-0.27‡	-0.37‡	0.24‡	-0.15‡	-	-0.43‡	-0.01	0.05
**a**	**TG**	0.18‡	0.23‡	0.29‡	0.36‡	-0.29‡	0.18‡	-0.41‡	-	0.11‡	0.04
**n**	**SBP**	0.18‡	0.16‡	0.11‡	0.05	-0.06	0.02	0.04	0.04	-	0.68‡
**s**	**DBP**	-0.03	-0.03	0.01	-0.06*	0.05*	0.00	0.07†	-0.02	0.74‡	-

**SAPPHIRe**	**N = 284**	**Japanese**									
**N = 747**		**BMI**	**WAIST**	**WHR**	**INS**	**GLUC**	**LDL**	**HDL**	**TG**	**SBP**	**DBP**

	**BMI**	-	0.83‡	0.31‡	0.64‡	-0.33‡	0.15*	-0.29‡	0.22‡	0.18‡	0.03
	**WAIST**	0.78‡	-	0.62‡	0.58‡	-0.28‡	0.17†	-0.24‡	0.20‡	0.09	-0.02
	**WHR**	0.38‡	0.73‡	-	0.30‡	-0.14	0.08	-0.13*	0.19‡	0.06	0.02
**C**	**INS**	0.54‡	0.51‡	0.31‡	-	-0.41‡	0.11	-0.37‡	0.32‡	0.19‡	0.09
**h**	**GLUC**	-0.24‡	-0.23‡	-0.15‡	-0.41‡	-	-0.07	0.35‡	-0.18†	-0.10	0.03
**i**	**LDL**	0.03	0.02	-0.03	0.01	0.05	-	-0.10	0.08	0.09	0.10
**n**	**HDL**	-0.23‡	-0.23‡	-0.16‡	-0.31‡	0.14‡	0.02	-	-0.41‡	-0.15*	-0.09
**e**	**TG**	0.33‡	0.30‡	0.25‡	0.40‡	-0.25‡	0.01	-0.37‡	-	0.21‡	0.12*
**s**	**SBP**	0.20‡	0.20‡	0.16‡	0.07	-0.04	0.01	-0.01	0.13‡	-	0.69‡
**e**	**DBP**	0.17‡	0.17‡	0.12‡	0.08*	-0.02	0.02	-0.02	0.14‡	0.84‡	-

* p < 0.05; † p < 0.01 ; ‡ p < 0.001 ; †† A negative correlation of GLUC and INS is result of the inverse squared transformation of GLUC

It is important to mention that each network had different exclusion criteria when recruiting participants: GENOA had excluded any case of HT secondary to other diagnoses or HT onset after age 60; HyperGEN had excluded type I diabetics, secondary hypertensives, or HT onset after age 60; SAPPHIRe had excluded participants with the following conditions: if they were using insulin or other prescription for diabetes, with cancer diagnosis, cirrhosis of the liver, terminal illness, and body mass index (BMI) > 35 kg/m^2^.

### Metabolic Syndrome, T2D and the VHA Definition

The categorical trait c-MetS was created employing the NCEP definition. c-MetS was defined by the presence of 3 or more of the following abnormalities in an individual: WAIST > 102 cm in men or > 88 cm in women, TG ≥ 150 mg/dl, HDL < 40 mg/dl in men or < 50 mg/dl in women, systolic blood pressure (SBP) ≥ 130 mm Hg and/or diastolic blood pressure (DBP) ≥ 85 mm Hg, or on treatment for HT, and GLUC ≥ 110 mg/dl or on treatment for diabetes [1]. The quantitative trait q-MetS was defined by the clustering patterns of 10 risk factors. The following risk factors were included in FA: BMI (kg/m^2^), WAIST (cm), waist-to-hip ratio (WHR), fasting insulin (INS, μU/ml), fasting GLUC (mg/dl), SBP and DBP, mm Hg), low density lipoprotein cholesterol (LDL, mg/dl), HDL (mg/dl) and fasting triglycerides (TG, mg/dl). To maintain consistency among the three networks, the minimum fasting time required was set at 8 hours.

Type 2 diabetes was defined by a fasting GLUC ≥ 126 mg/dl, or current use of hypoglycemic medication or insulin that was documented at examination in the clinic, or diabetes reported on questionnaires. An age of onset ≥ 40 years was also required to diagnose T2D [9].

In the FBPP pooled database, three important VHA variables were available from questionnaires: stroke or transient ischemic attacks, heart attack, and bypass or angioplasty. If any of these were reported in an individual, it was used to define the VHA status.

### Statistical analysis

The 10 risk variables of MetS were checked for normality and outliers. Each variable was adjusted for age, age^2^, age^3^, and field center within each gender-by-race-by-Network group. INS, TG, and HDL were transformed by natural logarithm to render them approximately normal. Likewise, GLUC was transformed as the inverse of the squared value (1/GLUC^2^). These transformations, together with standardization to zero mean and unit variance, prepared the data for FA. FA was performed by employing the *FACTANAL *function in S-plus version 6.1, Insightful Corp., Seattle. We applied an exploratory factor analysis where the extraction of the latent factors was performed based on the maximum likelihood estimation [10]. The statistical details of FA may be found elsewhere [11,12]. In short, FA explains the relationships among the risk variables in terms of a fewer number of underlying latent factors. The data were analyzed with and without Varimax rotation. When no rotation is applied, the first few factors can be considered the most important for MetS. Kaiser (1958) proposed the Varimax rotation that maximizes the sum of variances (of squares of loadings) for latent factors [13]. The two options provide ways of identifying the structure of the latent factors for MetS. One may consider "No rotation" for understanding how risk factors cluster to represent MetS; Varimax rotation may be used to identify distinct latent factors. This can be useful in genetic analyses [14]. Factor loadings are correlation coefficients between the original risk variables and the latent factors [12]. A loading ≥ 0.4 is interpreted as representing an important contribution of an original variable to the latent factor (marked in bold in Tables 6 and 7).

**Table 6:** Loadings of the Original Risk Factors in the Latent Factors by Network, Ethnicity (No Rotation)

**Rotation "NONE" Network/ Ethnicity**	Factor	**BMI**	**WAIST**	**WHR**	**INS**	**GLUC**	**LDL**	**HDL**	**TG**	**SBP**	**DBP**	**SS loadings**
**GENOA (African Americans)**	Factor 1	**0.75 **†	**0.68**	0.34	0.36	-0.26		-0.12	0.12	**0.75**	**0.48**	2.17
	Factor 2	**0.66**	**0.55**	0.18	0.28	-0.12		-0.14		**-0.66**	**-0.58**	1.66
	Factor 3		0.38	**0.92**	0.15	-0.16		-0.15	0.17			1.10
	Factor 4				**0.45**	-0.32	0.20	**-0.50**	**0.52**			0.87
	
**GENOA (Hispanics)**	Factor 1	**1.00**	**0.84**	0.33	**0.40**	-0.15		-0.17	0.13	0.16		2.07
	Factor 2		0.16	0.37		-0.21			0.28	**0.89**	**0.72**	1.61
	Factor 3		0.35	0.76	0.13	-0.13		-0.20	0.23	-0.29	-0.25	0.98
	Factor 4		-0.13	-0.22	0.11		0.18	-0.27	**0.85**			0.92
	
**GENOA (Whites)**	Factor 1	**1.00**	**0.92**	**0.57**	**0.59**	-0.33		-0.30	0.28	0.19		2.81
	Factor 2								0.12	**0.68**	**1.00**	1.50
	Factor 3				0.35	-0.25	0.19	**-0.53**	**0.47**			0.73
	Factor 4		0.27	**0.75**	0.12			-0.16				0.70

**HyperGEN (African Americans)**	Factor 1	**0.77**	**0.69**	0.37	0.35	-0.21		-0.11	0.15	**0.77**	**0.46**	2.21
	Factor 2	**0.64**	**0.58**	0.27	0.35	-0.16		-0.20	0.11	**-0.64**	**-0.60**	1.80
	Factor 3		0.36	**0.80**	0.28	-0.23		-0.25	0.30			1.05
	Factor 4				0.39	-0.34	0.18	**-0.51**	**0.54**			0.87
	
**HyperGEN (Whites)**	Factor 1	**0.96**	**0.97**	**0.61**	**0.53**	-0.33		-0.21	0.25	0.17		2.75
	Factor 2				0.11					**0.84**	**0.80**	1.37
	Factor 3				0.37	-0.21		**-0.65**	**0.56**			0.92
	Factor 4	-0.23	0.16	**0.58**					0.14			0.45

**SAPPHIRe (Chinese)**	Factor 1	0.38	**0.73**	**1.00**	0.32	-0.15		-0.17	0.25	0.16	0.12	1.93
	Factor 2	**0.53**	0.39		0.30	-0.14		-0.11	0.23	**0.77**	**0.78**	1.82
	Factor 3	**0.61**	**0.48**		**0.44**	-0.23		-0.21	0.18	**-0.45**	**-0.47**	1.36
	Factor 4		0.11		**-0.42**	0.36		0.37	**-0.49**			0.70
	
**SAPPHIRe (Japanese)**	Factor 1	**0.83**	**1.00**	**0.62**	**0.58**	-0.28	0.17	-0.24	0.20			2.63
	Factor 2	0.19			0.18			-0.14	0.15	**0.70**	**0.96**	1.54
	Factor 3			0.10	0.34	**-0.41**		**-0.60**	**0.50**	0.12		0.93
	Factor 4	**-0.52**		0.36	-0.24	0.18		0.11			0.27	0.59

†The loadings > = 0.40 are in bold.

**Table 7:** Loadings of the Original Risk Factors in the Latent Factors by Network, Ethnicity (Varimax Rotation)

Rotation "Varimax"	**GENOA**											
	
	**African Americans**				**Whites**				**Hispanics**			
	
**Factors / Variables**	Factor 1	Factor 2	Factor 3	Factor 4	Factor 1	Factor 2	Factor 3	Factor 4	Factor 1	Factor 2	Factor 3	Factor 4
BMI	**0.99** †	0.02	0.14	0.07	**0.97**	0.11	0.14	0.12	**0.99**†	0.05	-0.01	0.04
WAIST	**0.83**	0.03	0.19	**0.43**	**0.83**	0.10	**0.46**	0.15	**0.82**	0.07	0.01	**0.44**
WHR	0.28	0.06	0.22	**0.93**	0.38	0.06	**0.85**	0.15	0.29	0.08	-0.11	**0.88**
INS	0.38	0.01	**0.53**	0.10	**0.51**	0.03	0.18	**0.44**	0.39	0.04	0.15	0.12
GLUC	-0.21	-0.07	-0.38	-0.12	-0.28	-0.01	-0.09	-0.30	-0.14	-0.14	-0.18	-0.15
LDL	0.00	0.03	0.20	0.01	-0.03	0.04	-0.01	0.19	-0.03	0.04	0.20	0.01
HDL	-0.10	0.05	**-0.54**	-0.06	-0.20	0.05	-0.12	**-0.58**	-0.17	0.06	-0.31	-0.10
TG	0.04	0.01	**0.56**	0.08	0.20	0.10	0.07	**0.52**	0.13	0.07	**0.91**	-0.01
SBP	0.10	**0.99**	0.07	0.04	0.16	**0.68**	0.03	0.09	0.11	**0.94**	0.07	0.07
DBP	-0.04	**0.75**	0.02	0.02	-0.05	**0.99**	0.04	0.05	-0.01	**0.76**	0.04	0.04

SS Loadings	1.95	1.57	1.18	1.10	2.22	1.50	1.02	0.99	1.99	1.51	1.05	1.03
Cumulative Variance (%)	19.5	35.1	47	57.9	22.2	37.2	47.4	57.3	19.9	35.1	45.5	55.8

					
Rotation "Varimax"	**HyperGEN**											
					
	**African Americans**				**Whites**							
					
**Factors/Variables**	Factor 1	Factor 2	Factor 3	Factor 4	Factor 1	Factor 2	Factor 3	Factor 4				
				
BMI	**0.97**	0.04	0.24	0.09	**0.95**	0.06	0.17	0.14				
WAIST	**0.83**	0.03	0.25	**0.44**	**0.83**	0.02	0.15	**0.51**				
WHR	0.32	0.05	0.31	**0.81**	0.32	0.05	0.21	**0.75**				
INS	0.35	-0.02	**0.56**	0.17	**0.42**	0.11	**0.47**	0.15				
GLUC	-0.15	-0.02	**-0.45**	-0.13	-0.25	-0.07	-0.27	-0.13				
LDL	0.07	0.00	0.21	0.01	0.03	0.06	0.02	0.06				
HDL	-0.06	0.07	-0.06	-0.08	-0.08	0.05	**-0.68**	-0.05				
TG	0.02	0.02	**0.64**	0.12	0.09	0.07	**0.61**	0.13				
SBP	0.13	**0.99**	0.03	0.02	0.11	**0.85**	0.06	0.04				
DBP	-0.06	**0.76**	-0.06	0.02	-0.04	**0.80**	-0.02	0.05				
					
SS Loadings	1.90	1.56	1.54	0.93	1.97	1.40	1.23	0.90				
Cumulative Variance (%)	19.0	34.6	50.1	59.3	19.7	33.7	45.9	54.9				
					
Rotation "Varimax"	**SAPPHIRe**											
					
	**Chinese**				**Japanese**							
**Factors / Variables**	Factor 1	Factor 2	Factor 3	Factor 4	Factor 1	Factor 2	Factor 3	Factor 4				
					
BMI	**0.48**	**0.48**	0.08	**0.58**	**0.94**	0.06	0.25	0.24				
WAIST	**0.80**	0.38	0.08	0.35	**0.66**	-0.01	0.17	**0.73**				
WHR	**0.93**	0.18	0.11	-0.29	0.12	0.00	0.13	**0.71**				
INS	0.25	**0.69**	0.01	0.15	**0.49**	0.11	**0.48**	0.25				
GLUC	-0.07	**-0.47**	0.00	0.00	-0.20	0.03	**-0.47**	-0.08				
LDL	-0.01	-0.01	0.02	0.07	0.09	0.10	0.09	0.13				
HDL	-0.08	**-0.46**	0.01	0.03	-0.10	-0.09	**-0.65**	-0.09				
TG	0.12	**0.60**	0.12	-0.07	0.05	0.12	**0.54**	0.10				
SBP	0.09	0.03	**0.90**	0.11	0.10	**0.70**	0.16	0.01				
DBP	0.05	0.05	**0.92**	0.10	-0.05	**0.99**	0.00	0.04				
					
SS Loadings	1.85	1.68	1.68	0.60	1.64	1.53	1.32	1.20				
Cumulative Variance (%)	18.5	35.3	52.0	58.0	16.4	31.6	44.8	56.8				

†The loadings > = 0.40 are in bold.

Odds ratios, prevalence rates, and their confidence intervals were estimated by utilizing the *FREQ *and *LOGISTIC *regression procedures of SAS. Means and standard deviations were estimated with SAS software v. 9.0., SAS Institute, NC [15].

## Results

Mean age of participants ranged from 48 years in the HyperGEN African Americans to 58 years in the GENOA African Americans (Tables 1, 2, 3). Mean BMI and WAIST were higher for African Americans, Hispanics, and Whites than for Japanese and Chinese. A mean of approximately 130 mm Hg with a standard deviation of 20 mm Hg was evident for SBP across the networks and ethnicities.

### c-MetS

**Figure 1:**
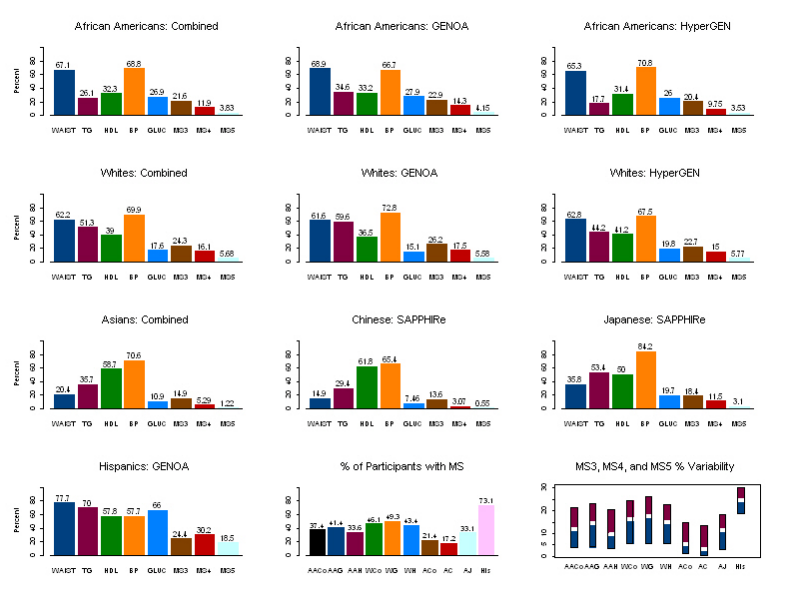
Percent of Participants by Network and Ethnicity that Pass Thresholds per Each Risk Factor as Defined by NCEP and Percent of Participants that had 3, 4, and 5 MetS Risk Factor Combinations Beyond the NCEP Thresholds. **Footnote**. MS3, MS4 and MS5: three, four and five risk factors beyond the NCEP thresholds; AACo-African Americans combined; AAG-Genoa African Americans; AAH-HyperGEN African Americans; WCo-Whites combined; WG-GENOA Whites; WH-HyperGEN Whites; ACo-Asians combined; AC-Chinese Asians; AJ-Japanese Asians; His-Hispanics

Among African Americans, twice as many were hypertriglyceridemic in GENOA than in HyperGEN, even though they were very comparable for all other MetS risk factors. This difference contributed to a relatively higher percentage of c-MetS in GENOA African Americans (41%) than in HyperGEN African Americans (34%). Whites in both networks were similar with respect to all NCEP thresholds (Figure 1).

The percentages of participants beyond the NCEP thresholds differed by ethnicity. The percentage of Japanese above the NCEP threshold for WAIST was half that of Hispanics, African Americans, or Whites, but twice that of the Chinese. The percentage of Japanese participants above the NCEP threshold for TG was similar to that in Whites, but about twice as large as in the Chinese; twice as many Whites were above the TG threshold than African Americans. The percentage of Japanese above the GLUC threshold, or on hypoglycemic medications, was similar to that in Whites, but about 3 times lower than in Hispanics and about 3 times higher than in the Chinese sample. The primary risk factors for c-MetS were WAIST, HDL, and blood pressure (BP) in African Americans, Hispanics, and Whites; TG had smaller weight in African Americans, but higher in Hispanics and Whites; in the Japanese, TG, HDL, BP, and to a smaller degree WAIST; and in the Chinese, HDL, BP, and less of TG. Hispanics also had a higher contribution of GLUC. Hispanics had a total of 73% of participants with at least 3 risk factors beyond the thresholds (MS3: 24%; MS4: 30%; MS5: 19%). Levels lower than the NCEP thresholds were more frequent in the Chinese and in the Japanese samples than in Hispanics, African Americans, or Whites. As expected, all networks and ethnicities selected had a high percentage of participants above the NCEP threshold for SBP/DBP or using hypertensive medications. For a better comparison, similar ethnicities were combined across networks (Figure 1).

Performing logistic regression on c-MetS risk factors, by using dummy variables defined as 1 if a risk factor was equal or beyond the NCEP thresholds, 0 otherwise, showed significant differences among ethnicities for most of the 5 NCEP MetS risk factors (Results not shown). Looking at the q-MetS patterns, Asians had more a contribution of hypertension and dyslipidemia. Whites and African Americans showed a classical MetS as a combination of obesity, dyslipidemia, and hypertension, much like the Hispanics (Figure 1).

### c-MetS and T2D

**Figure 2:**
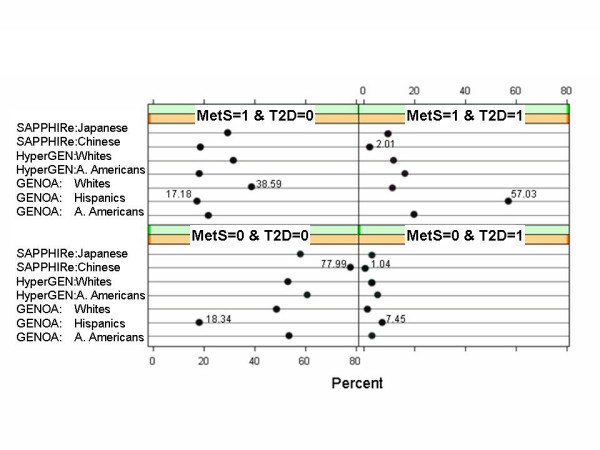
Frequency of Subjects by Network Within Ethnicity Given MetS and T2D Affection Status.

The presence of c-MetS was associated with T2D. This association was highly expressed in the Hispanics, where c-MetS and T2D were present simultaneously in 57% of the sample. The lowest percentage of participants with both c-MetS and T2D was 2% in the Chinese (Figure 2). All the association tests between c-MetS and T2D were significant with p-values < 0.0001 (Table 4). The odds of having T2D were 10.8 higher (8.3 – 14.1 a 95% odds CI) in GENOA African Americans if participants had c-MetS. The odds of having T2D when having c-MetS for Chinese and Japanese were about 5 with 95% odds CI (3.1 – 11.0) and (2.8 – 8.8), respectively.

**Table 4:** Association Between MetS and T2D in Networks Within Ethnicity

	**Odds Ratio**	**Prevalence Ratio**
**Network: Ethnicity**	**(95% Confidence Interval)**	**(95% Confidence Interval)**
**GENOA: African Americans**	10.8 (8.3 – 14.1)	6.2 (4.9 – 7.8)
**GENOA: Hispanics**	7.3 (5.8 – 9.2)	2.5 (2.2 – 2.8)
**GENOA: Whites**	8.0 (5.3 – 12.2)	6.5 (4.4 – 9.6)
**HyperGEN: African Americans**	9.7 (7.6 – 12.4)	5.7 (4.6 – 5.9)
**HyperGEN: Whites**	6.7 (4.9 – 9.1)	5.1 (3.9 – 6.8)
**SAPPHIRe: Chinese**	5.8 (3.1 – 11.0)	5.4 (2.9 – 9.9)
**SAPPHIRe: Japanese**	5.0 (2.8 – 8.8)	4.0 (2.4 – 6.6)

### VHA

The highest prevalence of VHA events was 17% in Hispanics, followed by the HyperGEN Whites with 15%; the lowest prevalence was 4% in the Chinese. The rest of the groups had intermediate VHA prevalence: GENOA African Americans, 10%; GENOA Whites, 10%; HyperGEN African Americans 13%; and Japanese 7%.

### q-MetS

Table 5 presents correlation matrices and sample sizes for 10 risk variables for MetS. Each upper or lower triangle of a matrix corresponds to correlation coefficients and the associated significance levels for a specified network and ethnicity. All networks and ethnicities studied showed a high correlation of BMI with WAIST (from 0.78 in the Chinese to 0.9 in the GENOA Whites and the HyperGEN African Americans), and a lower correlation of BMI with WHR (from 0.31 in the SAPPHIRe Japanese to 0.51 in the GENOA Whites). INS was correlated with BMI, WAIST, GLUC, and TG, but displayed significantly lower correlation with WHR. As expected TG was negatively correlated with HDL. SBP and DBP were highly correlated to each other (from 0.68 in the GENOA Whites to 0.84 in the SAPPHIRe Japanese). These patterns of correlations were likely to shape the latent factors created by analyzing the 10 MetS risk variables investigated.

### q-MetS with no Rotation

Tables 6 and 7 illustrate how groups of the original risk variables contribute (load) on latent factors from FA with and without Varimax rotation. The sums of squares of loadings (SS Loadings) correspond to the proportion of the variance of the original variables explained by each latent factor identified. When no rotation was performed on the factors, the main factor of African Americans was composed of BMI, INS, SBP, DBP, WAIST, and WHR. In GENOA Hispanics and Whites, BMI, INS, WAIST, and WHR contributed in the first factor. For the Chinese, the first factor loaded only obesity and explained 19% of the original variance; the third factor loaded primarily obesity, INS, and negative SBP and DBP, and explained about 14% of the original variance. The blood pressure components loaded in general on a separate factor with the exception of African Americans where the loading tended to be on the first factor. Lipids also contributed on a separate factor.

### q-MetS with Varimax Rotation

After performing the Varimax rotation, Factor 1 in the GENOA African Americans explained about 20% of the variance in the original 10 risk variables. Four factors explained from 55% of the variance in the original risk variables in the HyperGEN Whites to 60% in the HyperGEN African Americans. The four factors identified were not identical among the ethnicities in each network. Factor 1 loaded essentially BMI, WAIST, and INS in African Americans, Hispanics, Whites, and Japanese, but less BMI and INS compared to WAIST and WHR in Chinese. SBP and DBP contributed in a separate factor for each network and ethnicity. TG, HDL, INS, and GLUC contributed in a separate latent factor. The remaining fourth factors loaded WAIST and WHR in most of the networks' ethnicities, but in the Chinese loaded BMI and WAIST, with negative loading for WHR.

## Discussion

One of the major contributors to MetS, as can be seen in Figure 1, was high BP. About 70% of African Americans, Whites, or Asians, and 58% of Hispanics sampled had BP above the NCEP threshold or used anti-HT medications. These findings coincided with the main ascertainment in the sampled populations, reflecting the main goal of FBPP, to study the genetic causes of high blood pressure. The ascertainment schemes within each network may have played a role in the observed associations of the features of MetS and the prevalence of c-MetS. However, the characteristics described in the results stress that there are important ethnic differences, which need to be taken into consideration when evaluating / diagnosing MetS.

If we compare the prevalence of MetS in our study and a 23–24% of U.S. MetS prevalence reported by Ford et al. (2004) using data from the National Health and Nutrition Examination Survey (NHANES), it is evident that our US samples have a higher prevalence of MetS than the general US population [16]. In our study of 3,867 African Americans, 3,466 Whites, 2,211 Asians and 1,799 Hispanics, 37%, 46%, 21%, and 73%, respectively, were classified with c-MetS. This trend emphasizes the fact that selection for hypertension in most cases was associated with higher prevalence of MetS. Another example emphasizing that selection for a disorder part of the MetS, increases the prevalence of MetS, comes from a multinational study, Genetic Epidemiology of Metabolic Syndrome Project. This study has revealed a prevalence of 76% of MetS out of 1,436 participants, as result of selecting for atherogenic dyslipidemia [17].

The prevalence of MetS was comparable across Networks within the same ethnicity. However, there are ethnic differences in the prevalence of MetS. Prevalence of c-MetS is high in GENOA Hispanics (Figure 1). They also show high association of c-MetS with T2D (Figure 2). Although we believe that the prevalence of MetS is influenced by selection for type 2 diabetes, these results are in accordance with a large body of literature that illustrates that Hispanics have a trend for being more susceptible to MetS. Simon et al (2003) have reported that the prevalence of T2D was approximately two times higher among Hispanics than non-Hispanics [18]. McNeely and Boyko (2004) have reported that odds ratios for diabetes, compared to Whites, were 1 for Asians, 2.3 for African Americans, 2 for Hispanics, 2.2 for Native Americans, and 3.1 for Pacific Islanders [19]. Sanchez-Castillo et al. (2004) reported that in excess of 50% of adult population in Mexico are overweight and obese [20]. Furthermore, in our data, we found that the VHA events were highest in Hispanics. Our findings are in accord with the literature reporting that Mexican Americans had a 70% greater risk of cardiovascular mortality, and a 60% greater risk of coronary heart disease mortality than non-Hispanic Whites [21]. A higher incidence of hospitalized myocardial infarction in Mexican Americans than non-Hispanic Whites was also reported [22].

Conversely, Asians (and especially the Chinese) are leaner than others. We recognize that SAPPHIRe exclusion criteria biased the obesity findings. They also had lower T2D prevalence (Figure 2), because specifically the treated type 2 diabetics were excluded earlier than the clinical visit. They had lower prevalence of c-MetS. It is suggested that the NCEP criteria for obesity may not be suitable for the Japanese [23]. Tan et al. (2004) suggested that the NCEP definition of MetS underestimates its prevalence in Asian populations, because it embodies an unsuitable threshold of central obesity for Asians [24]. For example, in the FBPP Chinese sample (which represented individuals of Chinese origin living in Taiwan), if one would have lowered the threshold for WAIST as Tan et al. (2004) suggested, the prevalence of c-MetS in them would have increased.

Among African Americans and Hispanics, men had significantly lower odds of having c-MetS than women (Results not shown). Other authors have concluded that African-American women and Hispanic men and women have the highest prevalence of MetS. They attributed this to higher BP, obesity, and diabetes in African Americans, and the high prevalence of obesity and diabetes in Hispanics [25]. In the FBPP, more Whites had TG and HDL beyond the NCEP threshold as compared to African Americans.

In our study, each of the ethnicities considered showed significant MetS and T2D associations. Young et al. (2003), in a longitudinal cohort study of 429,918 veterans with diabetes, found that African Americans and Native Americans had a higher odds ratio (1.3 and 1.5 respectively) for having early diabetic nephropathy than Whites [26]. In the FBPP, the Hispanic sample exhibits a high occurrence of MetS along with T2D (57%) in association with a constellation of several risk factors for MetS beyond the NCEP thresholds. Our data (Figure 2), demonstrate also a small group of subjects with T2D, not classified as having MetS. This group is intriguing, because three or more risk factors are under the NCEP threshold, and it represents a deviation from the general notion that a cluster of risk factors of MetS may lead to T2D development. Is it possible that the scale for classifying T2D is error prone? Is there any genetic factor in this group that affects GLUC levels in the blood, without interfering with obesity and dyslipidemia pathways? A genetic analysis of this group in contrast with one having concurrently MetS and T2D, may identify important genetic differences related to MetS.

Four independent factors were identified when factor analysis was performed with Varimax rotation. Their pattern was very similar in African Americans, Hispanics, Whites, and Japanese, but not entirely so in Chinese. BMI, WAIST, and INS contributed together mainly in a factor labeled by us as "Obesity-INS." SBP and DBP contributed in a separate "BP" factor. A "Lipids-INS" factor was constructed mainly from contributions of LDL, HDL, TG, and INS. The last, "Central obesity" factor, was mostly an involvement of WAIST and WHR. These 4 factors were persistent also by gender in the HyperGEN data [14]. When no rotation was employed, the main MetS factor represented primarily a contribution of obesity together with INS in Hispanics, Whites, and Japanese; obesity and BP in African Americans; and obesity in Chinese. These patterns are quite important for a geneticist, because they show possible underlying trait combinations. The known interactions among traits grant ways to investigate the underlying genes, proteins and their substructures involved in these communications. For a clinician, the traits groupings shed light on the most important factors to be tackled when combating MetS. For the pharmacological research, these patterns can help in envisioning new medications intended to tackle the excess expression of risk variables in one, two, and/or three factors at once.

In general, our results about the structure of the factors, which reflect multivariate correlations of the variables studied, are supported by the literature. However, there are also differences that could be the result of variations in recruitment. In a study of Japanese Americans, it was found that visceral fat was a significant correlate of hypertension and independent of fasting INS [27]. In contrast, we found that correlations of WAIST/WHR with INS were highly significant in the Japanese, but not correlated with BP components.

In conclusion, patterns of the MetS were relatively similar across networks within ethnicity, but were statistically different among ethnicities. Overall, obesity was the most prominent compound risk factor expressed in both c-MetS and q-MetS. However, the degree of consistency in factor structures observed across ethnicities and networks is remarkable given that there are considerable differences in the Network-specific study designs. The notable exception of Hispanics in GENOA is quite understandable since the sample was also enriched for T2D. Thus, some of the differences especially in the prevalence of MetS are, at least in part, attributable to the study design differences. Nevertheless, the increase of MetS prevalence in our U.S. samples compared to the U.S. general population confirmed that there is an important link between HT and MetS. Together, our results underline that MetS is a compound phenotype, where obesity, dyslipidemia, and hypertension enable MetS. If we assume that obesity and dyslipidemia have separate biochemical pathways for their expression, it appears that the presence of INS in both latent obesity and lipids factors may be an indication that INS is an important contributor and possibly a connector of pathways in the development of MetS.

The reported findings will be useful if they lead to innovations. One application of these results can be the genetic analysis of the new MetS created data. It is well known that a categorical trait has less power in detecting genetic linkage as compared to a quantitative trait for a complex phenotype. Two types of q-MetS factor scores (with and without Varimax rotation) provide ample opportunity to discover quantitative trait loci for MetS. Parallel with this work, we have undertaken a detailed genetic analysis of the MetS factors that will be reflected in another publication (unpublished observations). Qualitative and quantitative characterization of MetS in the rich Family Blood Pressure Program pooled data will help in getting a better understanding of the genetic inheritance underlying MetS and its interaction with the environmental causes.

## List of Abbreviations Used

FBPP, Family Blood Pressure Program; NCEP, National Cholesterol Education Program; MetS, metabolic syndrome; c-MetS, qualitative MetS; q-MetS, quantitative MetS; OBS, obesity; T2D, type 2 diabetes; FA, factor analysis; VHA, vascular heterogeneous atherosclerotic events; HT, hypertension; BMI, body mass index; WAIST, waist circumference; WHR, waist to hip ratio; INS, insulin; GLUC, glucose; TG, triglycerides; LDL, low density lipoprotein cholesterol; HDL, high density lipoprotein cholesterol; SBP, systolic blood pressure; DBP, diastolic blood pressure; BP, blood pressure, CI-confidence interval.

## Competing interests

The author(s) declare that they have no competing interests.
